# Histone deacetylase 2 and N-Myc reduce p53 protein phosphorylation at serine 46 by repressing gene transcription of tumor protein 53-induced nuclear protein 1

**DOI:** 10.18632/oncotarget.1991

**Published:** 2014-05-20

**Authors:** Jeyran Shahbazi, Christopher J. Scarlett, Murray D. Norris, Bing Liu, Michelle Haber, Andrew E. Tee, Alice Carrier, Andrew V. Biankin, Wendy B. London, Glenn M. Marshall, Richard B. Lock, Tao Liu

**Affiliations:** ^1^ Children's Cancer Institute Australia for Medical Research, Sydney, Australia; ^2^ School of Biotechnology and Biomolecular Sciences, UNSW Science, University of New South Wales, Sydney, Australia; ^3^ School of Environmental and Life Sciences, University of Newcastle, Ourimbah, Australia; ^4^ Cancer Research Program, Garvan Institute of Medical Research, Sydney, Australia; ^5^ INSERM, U1068, CRCM ‘Stress cellulaire’, Marseille F-13009, France; ^6^ Wolfson Wohl Cancer Research Centre, University of Glasgow and Glasgow Royal Infirmary, Glasgow, United Kingdom; ^7^ Children's Oncology Group Statistics and Data Center and Boston Children's Hospital/Dana-Farber Cancer Institute, Boston, MA, USA; ^8^ Kids Cancer Centre, Sydney Children's Hospital, Sydney, Australia; ^9^ School of Women's and Children's Health, UNSW Medicine, University of New South Wales, Sydney, Australia

**Keywords:** N-Myc, HDAC2, p53, TP53INP1

## Abstract

Myc oncoproteins and histone deacetylases (HDACs) exert oncogenic effects by modulating gene transcription. Paradoxically, N-Myc induces p53 gene expression. Tumor protein 53-induced nuclear protein 1 (TP53INP1) phosphorylates p53 protein at serine 46, leading to enhanced p53 activity, transcriptional activation of p53 target genes and programmed cell death. Here we aimed to identify the mechanism through which N-Myc overexpressing p53 wild-type neuroblastoma cells acquired resistance to apoptosis. *TP53INP1* was found to be one of the genes most significantly repressed by HDAC2 and N-Myc according to Affymetrix microarray gene expression datasets. HDAC2 and N-Myc reduced *TP53INP1* gene expression by direct binding to the *TP53INP1* gene promoter, leading to transcriptional repression of *TP53INP1*, p53 protein de-phosphorylation at serine 46, neuroblastoma cell proliferation and survival. Moreover, low levels of TP53INP1 expression in human neuroblastoma tissues correlated with high levels of N-Myc expression and poor patient outcome, and the BET bromodomain inhibitors JQ1 and I-BET151 reduced N-Myc expression and reactivated TP53INP1 expression in neuroblastoma cells. These findings identify TP53INP1 repression as an important co-factor for N-Myc oncogenesis, and provide further evidence for the potential application of BET bromodomain inhibitors in the therapy of N-Myc-induced neuroblastoma.

## INTRODUCTION

Neuroblastoma, originating from precursor neuroblast cells in the sympathetic nervous system, is the most common malignancy of infancy and accounts for 15% of childhood cancer-related death [1]. Amplification of the *MYCN* oncogene and consequent over-expression of the N-Myc oncoprotein occur in 20-30% of primary untreated neuroblastoma tissues, and are highly correlated with advanced disease stage as well as poor patient prognosis [1, 2].

Myc oncoproteins induce tumor initiation and progression by modulating gene transcription. Myc dimerizes with MAX to form a Myc-MAX protein complex which directly binds to Myc-responsive element E-boxes at target gene promoters, leading to target gene transcription [3-5]. Myc oncoproteins also repress gene transcription by forming transcriptional repressor complexes with histone deacetylases (HDACs) at Sp1-binding sites of target gene promoters [6-9]. Identifying and further understanding the function of N-Myc target genes are important for developing better anticancer therapies.

HDACs are essential modulators of gene transcription, particularly of tumor suppressor genes [10]. The class I histone deacetylase HDAC2 is frequently overexpressed in human tumor tissues [6, 11, 12], and over-expression of HDAC2 induces tumor cell proliferation, blocks apoptosis and promotes tumor progression [13-16]. Factors which induce HDAC2 overexpression include N-Myc and c-Myc oncoproteins [6, 13].

The tumor protein 53-induced nuclear protein 1 (*TP53INP1*), also known as p53-dependent damage-inducible nuclear protein 1 (*P53DINP1*), is a stress-induced p53 target gene [17, 18]. TP53INP1 plays an important role in the phosphorylation of p53 protein at serine 46 (Ser-46), leading to enhanced p53 transcriptional activity, p53 target gene expression, cell growth arrest and apoptosis [17, 18]. Suppression of TP53INP1 has been shown to contribute to tumorigenesis of pancreatic cancer and lymphoma [19, 20].

Paradoxically, N-Myc oncoprotein induces gene transcription of the tumor suppressor gene p53 [21]. In this study, using microarray gene expression data, we observed that down-regulation of N-Myc or HDAC2 reactivated the expression of TP53INP1 in neuroblastoma cells. N-Myc and HDAC2 bound to the *TP53INP1* gene promoter, leading to transcriptional repression of TP53INP1, p53 protein de-phosphorylation at Ser-46, p53 inactivation and resistance to apoptosis.

## RESULTS

### Up-regulation of HDAC2 expression promotes survival of p53 wild type neuroblastoma cells

**Figure 1:**
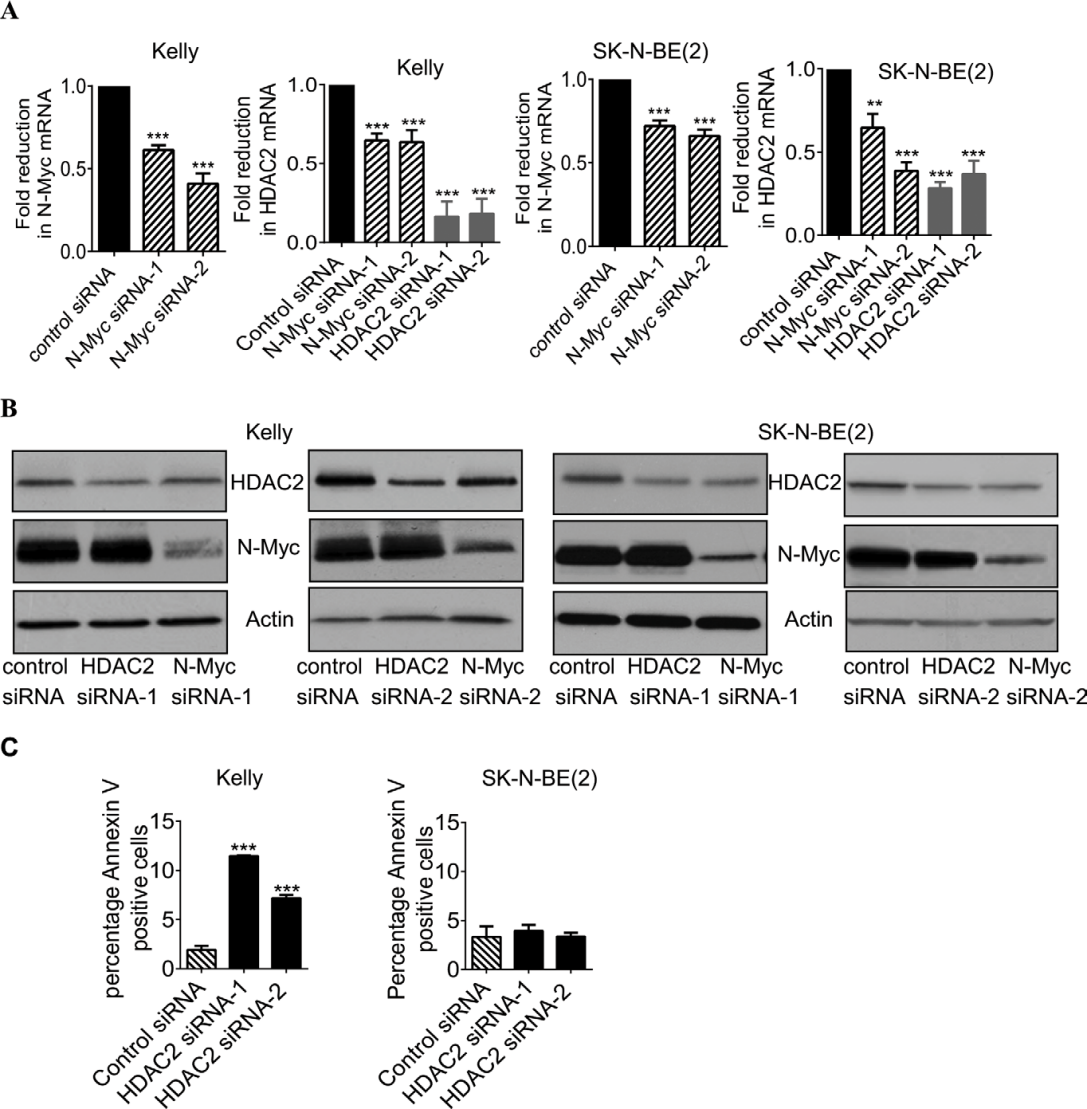
Up-regulation of HDAC2 expression promotes survival of p53 wild type neuroblastoma cells A and B, Kelly and SK-N-BE(2) neuroblastoma cells were transfected with scrambled control siRNA, N-Myc siRNA-1, N-Myc siRNA-2, HDAC2 siRNA-1 or HDAC2 siRNA-2 for 48 hours. RNA and protein were extracted from the cells and subjected to RT-PCR (A) and immunoblot (B) analyses of N-Myc and HDAC2 mRNA and protein expression. C, Kelly and SK-N-BE(2) cells were transfected with control siRNA, HDAC2 siRNA-1 or HDAC2 siRNA-2 for 72 hours. Cells were then stained with FITC-conjugated Annexin V and subjected to flow cytometry studies. The percentage of cells positively stained with Annexin V was analysed using FlowJo software. Error bars represent standard error. ** indicates *P* < 0.01 and *** indicates *P* < 0.001.

We have previously shown that HDAC2 induces cell proliferation, but not cell survival, in p53 mutant neuroblastoma cells [6]. To understand whether HDAC2 promoted survival in p53 wild type neuroblastoma cells, we first performed RT-PCR and immunoblot analysis of N-Myc and HDAC2 expression in the *MYCN* amplified Kelly (p53 wild type) and SK-N-BE(2) (p53 mutant) neuroblastoma cell lines after transfection with scrambled control siRNA, N-Myc siRNA (N-Myc siRNA-1 or siRNA-2) or HDAC2 siRNA (HDAC2 siRNA-1 or HDAC2 siRNA-2). As shown in Figures 1A and 1B, both N-Myc siRNA-1 and N-Myc siRNA-2 significantly reduced N-Myc mRNA and protein expression, and both HDAC2 siRNA-1 and HDAC2 siRNA-2 reduced HDAC2 mRNA and protein expression. While HDAC2 siRNAs showed no effect on N-Myc expression, knocking-down N-Myc expression with either one of the two N-Myc siRNAs reduced HDAC2 expression at both mRNA and protein levels.

To examine whether HDAC2 promoted cell survival, we stained Kelly and SK-N-BE(2) cells with Annexin V after the cells were transfected with control siRNA, HDAC2 siRNA-1 or HDAC2 siRNA-2 for 72 hours. Flow cytometry analysis showed that knocking-down HDAC2 expression increased the percentage of Kelly cells, but not SK-N-BE(2) cells, positively stained with Annexin V (Figure 1C). Taken together, the data suggest that N-Myc-mediated HDAC2 up-regulation promotes survival of the p53 wild type Kelly, but not the p53 mutant SK-N-BE(2), neuroblastoma cells.

### HDAC2 and N-Myc commonly down-regulate, and BET bromodomain inhibitors up-regulate, TP53INP1 expression

As a histone deacetylase, HDAC2 exerts its biological effects by modulating gene transcription. To understand how HDAC2 protected p53 wild type neuroblastoma cells against apoptosis, we examined Affymetrix microarray gene expression data, which were published previously [6], from neuroblastoma cells 32 hours after transfection with control siRNA or HDAC2 siRNA-1. The only gene which was significantly modulated by HDAC2 siRNA and potentially altered p53 function was *TP53INP1*. Interestingly, *TP53INP1* was also one of the genes most dramatically up-regulated by N-Myc siRNA-1 in the Affymetrix microarray gene expression study [6].

**Figure 2:**
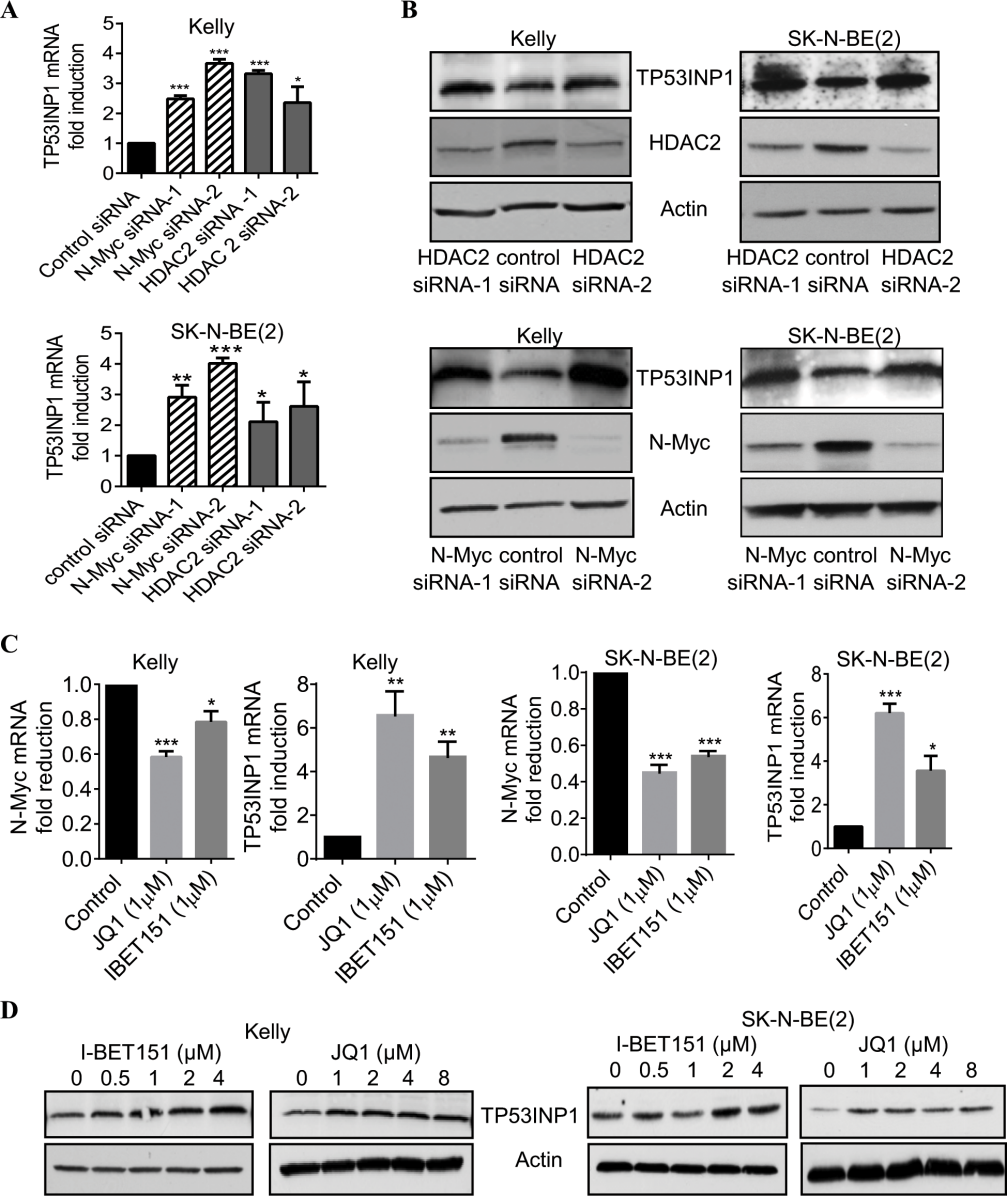
HDAC2 and N-Myc commonly repress, and BET bromodomain inhibitors up-regulate, TP53INP1 expression A and B, Kelly and SK-N-BE(2) neuroblastoma cells were transfected with scrambled control siRNA, N-Myc siRNA-1, N-Myc siRNA-2, HDAC2 siRNA-1 or HDAC2 siRNA-2 for 48 hours. RNA and protein were extracted from the cells and subjected to RT-PCR (A) and immunoblot (B) analyses of TP53INP1 mRNA and protein expression. C and D, Kelly and SK-N-BE(2) cells were treated with vehicle control or various doses of the BET bromodomain inhibitor I-BET151 or JQ1 for 48 hours, followed by RNA and protein extraction. C, N-Myc and TP53INP1 mRNA expression was analysed by RT-PCR. D, TP53INP1 protein expression was analysed by immunoblot. Error bars represent standard error. *, ** and *** indicate *P* < 0. 05, 0.01 and 0.001, respectively.

To validate the Affymetrix microarray data, we performed RT-PCR and immunoblot analyses of TP53INP1 expression in Kelly and SK-N-BE(2) neuroblastoma cells after down-regulation of N-Myc and HDAC2 using siRNA transfection. As shown in Figures 2A and 2B, both N-Myc siRNAs and HDAC2 siRNAs significantly increased TP53INP1 mRNA and protein expression in the p53 wild type Kelly and the p53 mutant SK-N-BE(2) neuroblastoma cells.

We next treated the neuroblastoma cells with the BET bromodomain inhibitor JQ1 or I-BET151, both of which have been shown to inhibit N-Myc gene transcription and expression [28]. RT-PCR analysis confirmed the down-regulation of N-Myc mRNA expression by these two BET bromodomain inhibitors in Kelly and SK-N-BE(2) cells (Figure 2C). Importantly, JQ1 and I-BET151 considerably induced TP53INP1 expression at both mRNA and protein levels (Figure 2D). The data therefore demonstrate that N-Myc and HDAC2 commonly down-regulate TP53INP1 expression, and this down-regulation can be reversed by treatment with BET bromodomain inhibitors.

### N-Myc and HDAC2 block p53 protein phosphorylation at Ser-46

**Figure 3:**
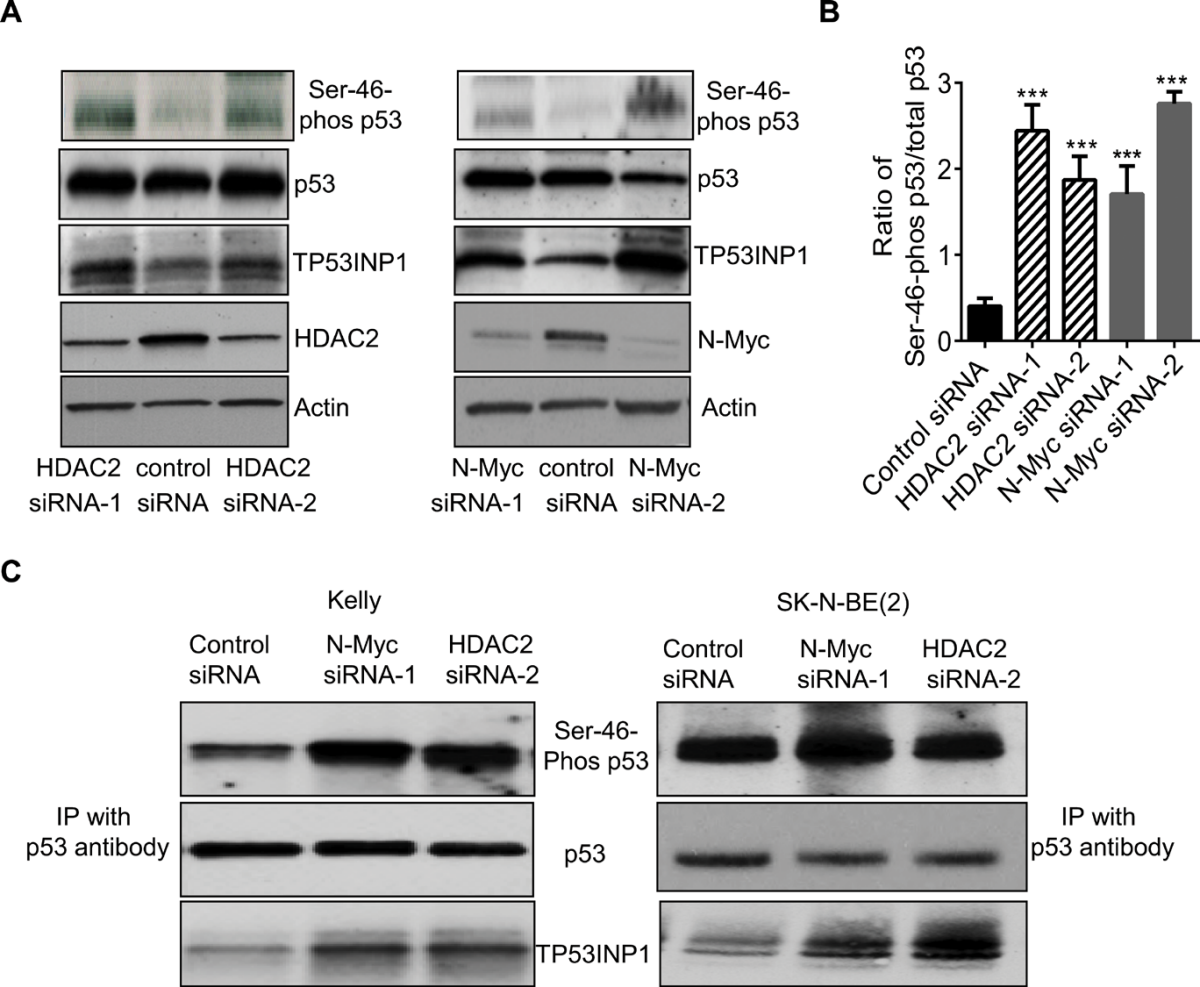
N-Myc and HDAC2 block p53 protein phosphorylation at Ser-46 A and B, Kelly neuroblastoma cells were transfected with control siRNA, N-Myc siRNA-1, N-Myc siRNA-2, HDAC2 siRNA-1 or HDAC2 siRNA-2 for 48 hours. A, protein was extracted from the cells and subjected to immunoblot analyses with anti-Ser-46-phosphorylated p53 (Ser-46-phos p53), anti-total p53 and anti-TP53INP1 antibodies. B, the ratio of Ser-46-phos p53 to total p53 was quantified. C, Kelly and SK-N-BE(2) cells were transfected with control siRNA, N-Myc siRNA-1 or HDAC2 siRNA-2 for 48 hours. Protein was extracted from the cells and immunoprecipitated (IP) with an anti-p53 antibody. The immunoprecipitated protein was subjected to immunoblot analyses with anti-Ser-46-phos p53, anti-total p53 and anti-TP53INP1 antibodies. Error bars represent standard error. *** indicates *P* < 0.001.

TP53INP1 is well-known to induce apoptosis by causing p53 protein phosphorylation at Ser-46, leading to p53 activation [17, 18]. Since N-Myc siRNAs and HDAC2 siRNAs up-regulated TP53INP1 expression, we next examined whether N-Myc and HDAC2 could modulate p53 protein phosphorylation at Ser-46. As shown in Figure 3A, siRNA-mediated knock-down of either N-Myc or HDAC2 expression significantly increased the level of p53 protein phosphorylated at Ser-46, in addition to up-regulating TP53INP1 protein expression, in the Kelly neuroblastoma cells. Quantification of the ratio of Ser-46-phosphorylated p53 to total p53 further confirmed the increased phosphorylation (Figure 3B).

We next extracted protein from Kelly cells after transfection with control siRNA, N-Myc siRNA-1 or HDAC2 siRNA-2, and performed protein co-immunoprecipitation assays with an anti-p53 antibody. As shown in Figure 3C, the anti-p53 antibody immunoprecipitated total p53 protein, Ser-46-phosphorylated p53 protein as well as TP53INP1 protein. Knocking-down N-Myc or HDAC2 expression with siRNAs had no effect on the level of total p53 protein immunoprecipitated by the anti-p53 antibody, increased the level of TP53INP1 protein immunoprecipitated by the anti-p53 antibody, and increased the level of Ser-46 phosphorylated p53 protein immunoprecipitated by the anti-p53 protein in the p53 wild type Kelly cells. By contrast, in SK-N-BE(2) cells with mutant p53, N-Myc siRNA and HDAC2 siRNA up-regulated TP53INP1 expression, but showed no effect on the expression level of Ser-46-phosphorylated p53 (Figure 3C). The data suggest that N-Myc and HDAC2 reduce p53 protein phosphorylation at Ser-46 by repressing TP53INP1 gene transcription in p53 wild type, but not p53 mutant, cells, and that TP53INP1 induces p53 protein phosphorylation by forming a protein complex with p53.

### Transcriptional repression of TP53INP1 protects neuroblastoma cells against apoptosis

**Figure 4:**
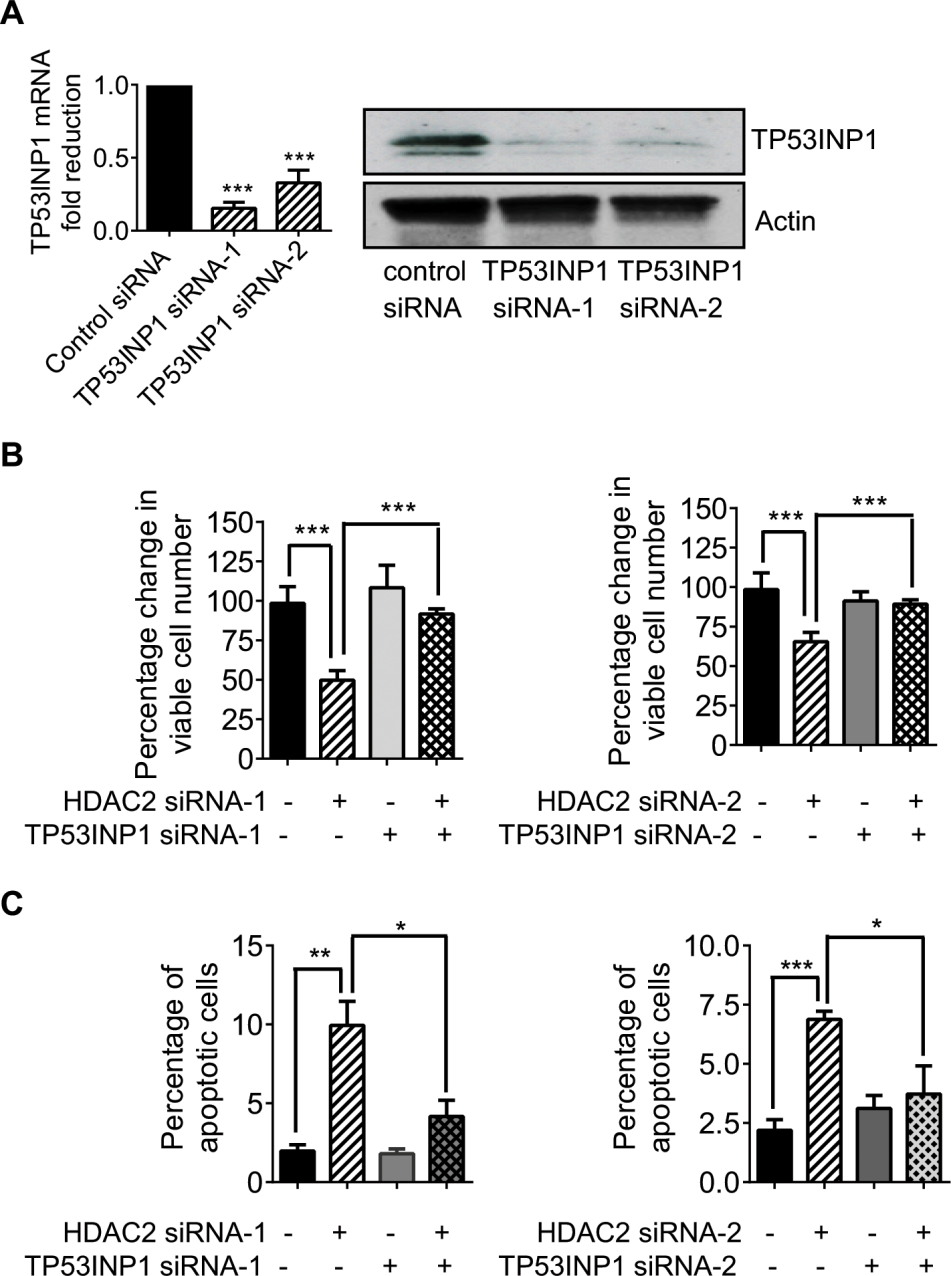
Transcriptional repression of TP53INP1 protects neuroblastoma cells against apoptosis A, Kelly cells were transfected with control siRNA, TP53INP1 siRNA-1 or TP53INP1 siRNA-2. TP53INP1 mRNA and protein expression was analysed by real-time RT-PCR and immunoblot. B and C, Kelly cells were transfected with control siRNA, HDAC2 siRNA-1, HDAC2 siRNA-2, TP53INP1 siRNA-1, TP53INP1 siRNA-2, HDAC2 siRNA-1 plus TP53INP1 siRNA-1, or HDAC2 siRNA-2 plus TP53INP1 siRNA-2. Seventy-two hours later, the cells were incubated with Alamar blue for Alamar blue assays (B) or stained with FITC-conjugated Annexin V for flow cytometry analysis of apoptotic cells (C). Error bars represent standard error. *, ** and *** indicate *P* < 0.05, 0.01 and 0.001, respectively.

As TP53INP1 is well-known to induce cancer cell apoptosis by promoting p53 protein phosphorylation at Ser-46 and hence activating this tumor suppressor [17, 18], we examined whether repression of TP53INP1 by HDAC2 could protect neuroblastoma cells against apoptosis. As shown in Figure 4A, transfection of the Kelly neuroblastoma cells with two independent TP53INP1 siRNAs led to a marked knock-down of TP53INP1 mRNA and protein expression. While knock-down of TP53INP1 on its own did not have significant effects on cell viability, knock-down of HDAC2 reduced the number of viable Kelly cells by approximately 50-60% (Figure 4B). Importantly, simultaneous knock-down of these two genes reversed the effects observed with HDAC2 siRNA alone (Figure 4B). Consistent with these results, flow cytometry analysis showed that HDAC2 down-regulation increased the percentage of Kelly cells positively stained with FITC-conjugated Annexin V, while TP53INP1 on its own had no detectable effects (Figure 4C). However, knock-down of TP53INP1 significantly reversed the HDAC2 siRNA-mediated increase in the percentage of Annexin V-positive cells. These data suggest that HDAC2-mediated transcriptional repression of TP53INP1 protects neuroblastoma cells against apoptosis in the p53 wild type cells.

### N-Myc and HDAC2 reduce TP53INP1 gene expression by direct binding to the TP53INP1 gene promoter

**Figure 5:**
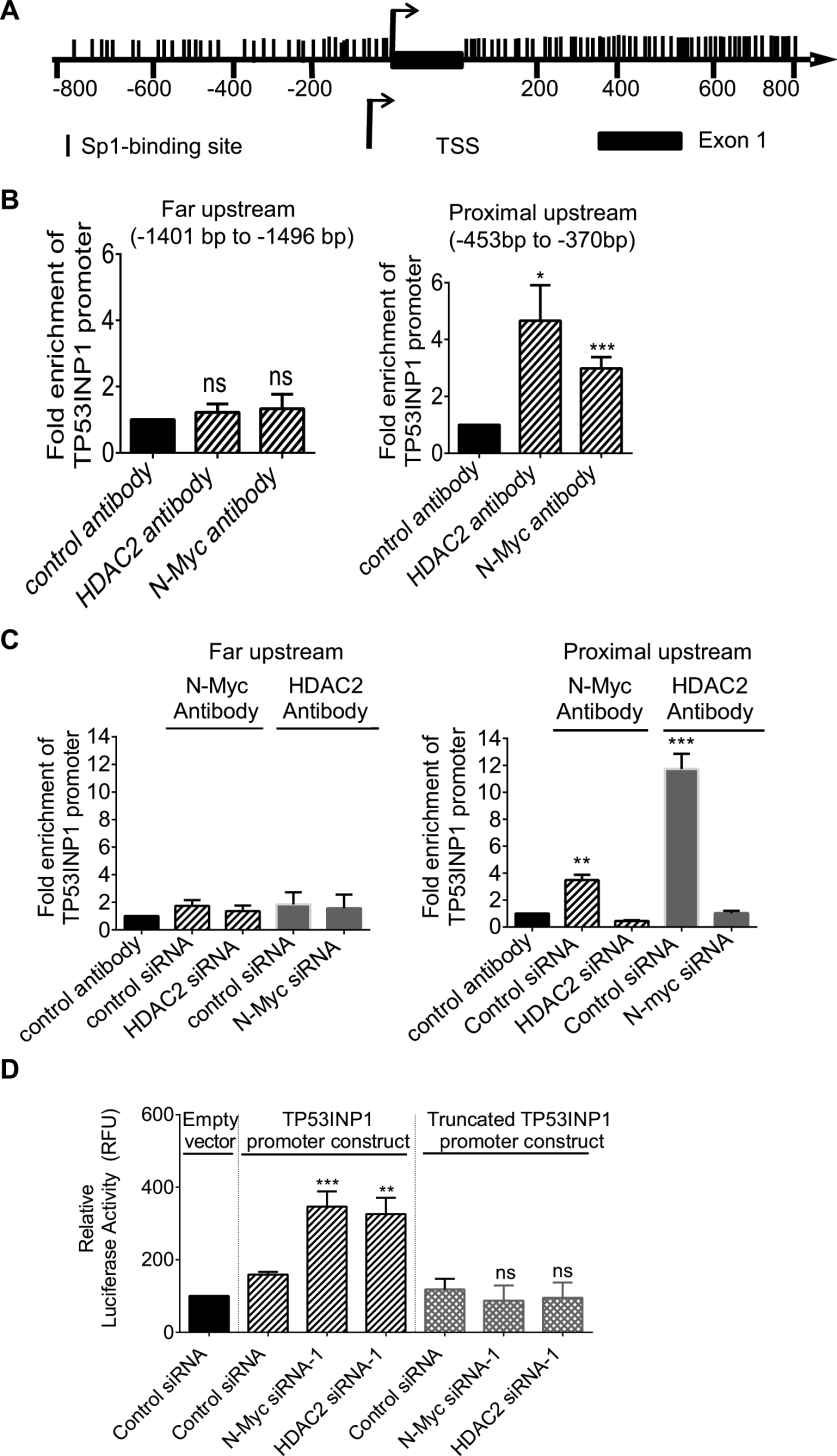
N-Myc and HDAC2 reduce TP53INP1 gene expression by direct binding to the TP53INP1 gene promoter A, schematic representation of the *TP53INP1* gene promoter. TSS represents the transcription start site and each vertical line represents an Sp1-binding site. B, ChIP assays were performed with a control IgG, an anti-N-Myc antibody or an anti-HDAC2 antibody and PCR with primers targeting a negative control region (-1401 bp to -1496 bp upstream of TSS) or proximal upstream region (-453 bp to -370 bp) of the *TP53INP1* gene promoter in Kelly cells. Fold enrichment of the *TP53INP1* gene promoter by the control IgG, the anti-N-Myc antibody or the anti-HDAC2 antibody was calculated by dividing PCR products from primers targeting the proximal upstream region by PCR products from primers targeting the negative control region. C, ChIP assays were performed with a control IgG or an anti-N-Myc antibody in Kelly cells 48 hours after transfection with control siRNA or HDAC2 siRNA-1, and performed with a control IgG or an anti-HDAC2 antibody in Kelly cells 48 hours after transfection with control siRNA or N-Myc siRNA-1. PCR and data analysis were performed as above. D, luciferase assays were performed in Kelly cells after co-transfection with control siRNA, N-Myc siRNA-1 or HDAC2 siRNA-1, together with Cypridina TK control construct plus pLightSwitch_Prom construct expressing empty vector, wild type or proximal upstream truncated *TP53INP1* gene promoter for 48 hours. Luciferase activities were measured with a LightSwitch Dual Assay System Kit, normalized according to Cypridina TK control construct, and expressed as percentage changes relative to samples transfected with control siRNA plus empty vector pLightSwitch_Prom construct. Error bars represent standard error. *, ** and *** indicate *P* < 0.05, 0.01 and 0.001 respectively, and ns indicates no significant difference.

N-Myc has been shown to recruit HDAC2 to Sp1-binding site-enriched regions of miR-183 and cyclin G2 gene promoters, leading to transcriptional repression of miR-183 and cyclin G2 [6, 29]. To understand whether N-Myc and HDAC2 could directly repress TP53INP1 gene transcription, we first analysed transcription factor binding sites at the TP53INP1 gene promoter with Gene-Regulation software (http://www.gene-regulation.com/pub/programs/alibaba2/index.html). Results showed that Sp1-binding sites were exceptionally enriched at -800bp to 0bp upstream of the TP53INP1 gene transcription start site as well as +0bp to +800bp in intron 1 (Figure 5A). We therefore performed chromatin immunoprecipitation (ChIP) assays with an anti-N-Myc antibody, an anti-HDAC2 antibody or a control IgG. PCR was carried-out with primers targeting the Sp1-binding site-enriched regions of the TP53INP1 gene promoter or a negative control region far up-stream of the TP53INP1 transcription start site. The ChIP assays showed that N-Myc and HDAC2 both bound to the proximal upstream region of the TP53INP1 gene (Figure 5B).

To investigate whether HDAC2 and N-Myc bind to the *TP53INP1* gene promoter dependent on each other, we performed ChIP assays with a control IgG or an anti-N-Myc antibody in Kelly cells after transfection with control siRNA or HDAC2 siRNA-1, and performed ChIP assays with a control IgG or an anti-HDAC2 antibody in Kelly cells after transfection with control siRNA or N-Myc siRNA-1. PCR analyses were then performed with primers targeting the far upstream control region or the proximal upstream region of the *TP53INP1* gene promoter. Results showed that knocking-down HDAC2 expression blocked N-Myc protein binding to the proximal upstream region of the TP53INP1 gene promoter, and that knocking-down N-Myc expression blocked HDAC2 protein binding to the proximal upstream region of the TP53INP1 gene promoter (Figure 5C). The data suggest that binding of N-Myc to the TP53INP1 gene promoter is dependent on HDAC2, and that binding of HDAC2 to the TP53INP1 gene promoter is dependent on N-Myc.

To further understand whether the binding of N-Myc and HDAC2 to the *TP53INP1* gene promoter region repressed *TP53INP1* gene transcription, we cloned wild type *TP53INP1* gene promoter (-945bp to +154bp relative to transcription start site) and control truncated *TP53INP1* gene promoter (-945bp to -645bp relative to transcription start site) into the pLightSwitch_Prom construct. Kelly cells were then co-transfected with control siRNA, N-Myc siRNA-1 or HDAC2 siRNA-1, together with Cypridina TK control construct plus the pLightSwitch_Prom construct expressing empty vector, the wild type or the truncated *TP53INP1* promoter. Luciferase assays showed that knocking-down N-Myc or HDAC2 expression significantly up-regulated luciferase activity of the wild type, but not the proximal upstream truncated, *TP53INP1* promoter construct (Figure 5D). The data indicate that N-Myc and HDAC2 reduce TP53INP1 expression by direct binding to the *TP53INP1* gene promoter and repressing *TP53INP1* gene transcription.

### Low levels of TP53INP1 expression in tumor tissues correlate with high levels of N-Myc expression and poor prognosis in neuroblastoma patients

**Figure 6:**
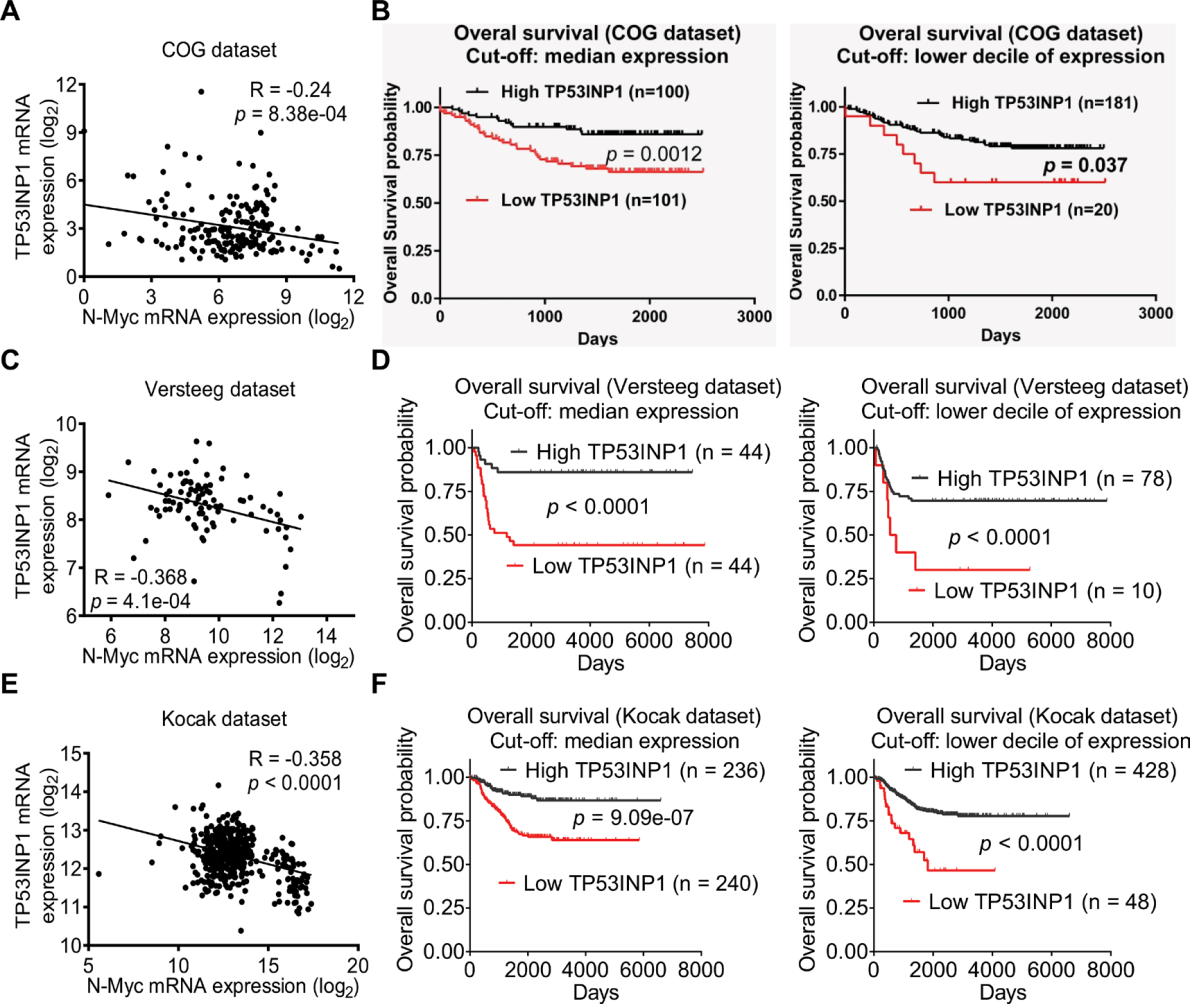
Low levels of TP53INP1 expression in tumor tissues correlate with high levels of N-Myc expression and poor prognosis in neuroblastoma patients. A, RT-PCR studies were performed in 201 primary human neuroblastoma from Children's Oncology Group (COG) with primers targeting TP53INP1 or N-Myc. Correlation between TP53INP1 expression and N-Myc expression in the tumor tissues was analysed by Pearson's correlation. B, Kaplan–Meier survival analysis was performed based on the level of expression of TP53INP1 in the 201 neuroblastoma patients. The level of expression was considered high or low in relation to the median or lower decile of TP53INP1 expression of all tumors analyzed. C and D, correlation between TP53INP1 expression and N-Myc expression was analysed in 88 and 476 human neuroblastoma samples in publically available microarray gene expression datasets of Versteeg (C) and Kocak (D). E and F, Kaplan–Meier survival analysis was performed based on the level of TP53INP1 expression in the 88 and 476 neuroblastoma patients in the Versteeg dataset (E) and the Kocak dataset (F). The level of expression was considered high or low in relation to the median or lower decile of TP53INP1 expression of the tumors analyzed.

To assess whether repression of TP53INP1 expression in neuroblastoma tissues can be used as a novel prognostic marker for poor prognosis and as a therapeutic target in neuroblastoma patients, we examined TP53INP1 gene expression in a cohort of 201 neuroblastoma cDNA samples from the Pediatric Oncology Group Neuroblastoma Biology Study [24]. Real-time RT-PCR studies showed that TP53INP1 mRNA expression negatively correlated with N-Myc mRNA expression in the 201 human neuroblastoma tissues (Figure 6A). Importantly, using the median or lower decile of TP53INP1 mRNA expression as the cut-off point, Kaplan-Meier analysis showed that low levels of TP53INP1 mRNA expression in tumor tissues were significantly associated with poor patient survival (Figure 6B). Similar results were obtained when the data were dichotomized using the lower or upper quartile as cut-off points (data not shown). Consistent with these findings, the publically available (http://r2.amc.nl) Versteeg and Kocak [25, 26] microarray gene expression datasets from 88 and 476 patients respectively, showed that TP53INP1 mRNA expression in human neuroblastoma tissues negatively correlated with N-Myc mRNA expression (Figures 6C & 6E), and that low levels of TP53INP1 mRNA expression in tumor tissues were significantly associated with poor patient outcome (Figures 6D & 6F).

## DISCUSSION

We have previously demonstrated that N-Myc and HDAC2 commonly suppress the expression of the cyclin G2 gene by forming a transcriptional repressor complex at an Sp1-binding site-enriched region of the cyclin G2 gene promoter [6]. More recently, N-Myc has been shown to recruit HDAC2 to the miR-183 promoter, leading to transcriptional suppression of miR-183 [29]. In the current study, we have confirmed that N-Myc up-regulates HDAC2 expression in p53 mutant and p53 wild type neuroblasotma cells, and have identified TP53INP1 as a major downstream target of these transcriptional regulators. In this regard, bioinformatic analysis showed that the *TP53INP1* gene promoter region is exceptionally enriched of Sp1-binding sites, while ChIP and luciferase assays demonstrated that N-Myc and HDAC2 both bind to the *TP53INP1* gene promoter region to enhance TP53INP1 promoter activity. Taken together, our data indicate that N-Myc and HDAC2 commonly repress *TP53INP1* gene transcription by forming a transcriptional repressor complex at the *TP53INP1* gene promoter.

TP53INP1 is known to form a protein complex with homeodomain-interacting protein kinase-2 (HIPK2) or PKCδ kinases, leading to p53 protein phosphorylation at Ser-46, induction of p53AIP1 gene transcription, p53 binding to target gene promoters, and finally, p53 target gene expression and apoptosis [17, 18, 30]. On the other hand, HDAC2 induces cancer cell proliferation in p53 mutant neuroblastoma and pancreatic cancer cells [6], while sumoylation of HDAC2 controls p53 deacetylation and restricts apoptosis following genotoxic stress [31, 32]. However, the mechanism by which HDAC2 regulates p53-DNA binding activity and modulates p53 transcriptional activities is largely unknown [33]. In this study, we have shown that HDAC2 suppresses TP53INP1 expression and consequently reduces p53 protein phosphorylation at Ser-46, that up-regulation of HDAC2 protects p53 wild type neuroblastoma cells against apoptosis, and that apoptosis due to loss of HDAC2 expression can be blocked by simultaneous depletion of TP53INP1 from the cell. The data suggest that HDAC2 protects p53 wild type neuroblastoma cells against apoptosis by suppressing TP53INP1 gene expression, leading to p53 protein de-phosphorylation at Ser-46 and p53 protein inactivation. Additionally, N-Myc is well-known to up-regulate p53 mRNA and protein expression by direct binding to the p53 gene promoter and activating p53 gene transcription [21]. Our data indicate that N-Myc inactivates p53 protein function and protects cells against apoptosis by suppressing TP53INP1 gene transcription and consequently blocking p53 protein phosphorylation at Ser-46.

While HDAC inhibitors have shown anticancer effects in cancer patients, the BET bromodomain inhibitors JQ1 and I-BET151 are among the most promising novel anticancer agents identified to date [34-37]. JQ1 and I-BET151 block the function of BET bromodomain proteins BRD1, BRD2, BRD3 and BRD4 by displacing them from active histones [35]. Since their discovery in 2010, JQ1 and I-BET151 have shown considerable anticancer effects against leukemia [37-39], lymphoma [36], myeloma [34], lung cancer [40] and neuroblastoma [28] *in vitro* and *in vivo*, through blocking the transcription of both c-Myc and N-Myc. Consequently, pharmaceutical companies are racing to test JQ1 and I-BET151 in cancer patients [41]. Our previous Affymetrix microarray data show that the HDAC inhibitor Trichostatin A up-regulates TP53INP1 gene expression, and the current study reveals that treatment of neuroblastoma cells with JQ1 or I-BET151 considerably reduces N-Myc expression and up-regulates TP53INP1 expression, and that a low level of TP53INP1 expression in human neuroblastoma tissues correlates with a high level of N-Myc expression and poor prognosis. The data suggest that a low level of TP53INP1 expression in human neuroblastoma tissues can be used as a marker for poor prognosis and for therapeutic application of the BET bromodomain inhibitors in the patients.

In summary, this study demonstrates that a novel pathway, involving transcriptional repression of TP53INP1, reduction in p53 protein phosphorylation at Ser-46 and consequent p53 protein inactivation, contributes to N-Myc and HDAC2-mediated cancer cell survival. Moreover, repression of TP53INP1 in human neuroblastoma tissues is a marker for poor patient prognosis, and BET bromodomain inhibitors reactivate TP53INP1 expression in cancer cells. These findings therefore identify TP53INP1 repression as an important co-factor for N-Myc oncogenesis, and provide further evidence for the potential application of BET bromodomain inhibitors in the therapy of N-Myc-induced neuroblastoma.

## METHODS

### Cell culture

Human neuroblastoma SK-N-BE(2) and Kelly cells were cultured in Dulbecco's modified Eagle's medium (supplemented with 10% fetal calf serum) and RPMI 1640 (supplemented with 10% fetal calf serum and 1% L-glutamine), respectively [7, 22].

### Small interfering RNA (siRNA) and plasmid transfection

Cells were transfected with siRNAs or plasmids using Lipofectamine 2000 reagent (Invitrogen, Carlsbad, CA, USA) as we have described previously [7, 22]. Validated scrambled control siRNA, siRNAs specifically targeting N-Myc, HDAC2 or TP53INP1 were purchased from Qiagen (Qiagen, Hamburg, Germany) and/or Ambion (Ambion, Austin, TX, USA).

### Quantitative real-time RT–PCR analysis

Following siRNA transfections, RNA was extracted from cells using PureLink RNA Mini kit (Invitrogen) according to the manufacturer's instructions. RNA samples were then quantified using a Nanodrop spectrophotometer (Nanodrop, Wilmington, DE, USA). Synthesis of cDNA from RNA samples was carried out using M-MLV Reverse Transcriptase (Sigma, St Louis, MO, USA). Real-time RT-PCR was performed as we have previously described [7-9].

### Immunoblot analysis

For the analysis of protein expression by immunoblot, cells were lysed, protein extracted and separated by gel electrophoresis. After western transfer, membranes were probed with an anti-N-Myc (1:1000, Santa Cruz Biotech, CA, USA), anti-HDAC2 (1:2000, Santa Cruz Biotech), anti-TP53INP1 (1:500, Abcam, Cambridge, MA, USA), anti-Ser-46- phosphorylated p53 (1:250, Cell Signaling, Danvers, MA, USA) or anti-p53 antibody (1:500, Cell Signaling). Protein bands were visualized with SuperSignal (Pierce, Rockford, IL, USA). The membranes were lastly re-probed with an anti-actin antibody (Sigma) as loading controls.

### Immunoprecipitation assays

Kelly neuroblastoma cells were transfected with control siRNA, N-Myc siRNA or HDAC2 siRNA for 48 hours. Cellular protein was extracted and incubated overnight with 2 mg of anti-p53 antibody. Eluted protein was immunoblotted with anti-Ser-46-phosphorylated p53, p53 and TP53INP1 antibodies.

### Chromatin immunoprecipitation (ChIP) assays

ChIP assays were performed with an anti-N-Myc antibody, anti-HDAC2 antibody or control antibody, and PCR performed with primers targeting a negative control region (-1401 bp to -1496 bp upstream of transcription start site) or proximal upstream region (-453 bp to -370 bp upstream of transcription start site) of the *TP53INP1* gene promoter. Fold enrichment of the proximal upstream region of the *TP53INP1* gene promoter by the anti-N-Myc antibody or the anti-HDAC2 antibody was calculated by dividing PCR signal from the proximal upstream region of the *TP53INP1* gene promoter by PCR signal from the negative control region.

### Luciferase assays

Modulation of *TP53INP1* promoter activity by N-Myc and HDAC2 was analysed by luciferase assays. Wild type *TP53INP1* gene promoter (-945bp to +154bp relative to transcription start site) and control truncated *TP53INP1* gene promoter (-945bp to -645bp relative to transcription start site) were cloned into the pLightSwitch_Prom construct(SwitchGear Genomics, Menlo Park, CA, USA). Kelly neuroblastoma cells were co-transfected with control siRNA, N-Myc siRNA-1 or HDAC2 siRNA-1, together with Cypridina TK control construct plus the pLightSwitch_Prom construct expressing empty vector, the wild type or the truncated *TP53INP1* promoter for 48 hours. Luciferase activities were measured with a LightSwitch Dual Assay System Kit (SwitchGear Genomics), normalized according to Cypridina TK control construct according to the manufacturer's instructions, and expressed as percentage changes relative to control siRNA and empty vector pLightSwitch_Prom construct transfected samples.

### Alamar blue assays

Alamar blue assays were performed as previously described [23]. Briefly, cells were transfected with siRNAs in 96 well plates. Seventy-two hours after siRNA transfections, cells were incubated with Alamar blue (Invitrogen), and the plates were read on a microplate reader at 570/595 nm. Results were calculated according to optical density absorbance units and expressed as percentage change in the number of viable cells using mitochondrial metabolic activity as a surrogate indicator.

### Flow cytometry studies

Seventy-two hours after siRNA transfections, cells were harvested, washed and then stained with FITC-conjugated Annexin V (FITC Apoptosis Detection Kit, BD Biosciences, San Jose, CA, USA). Flow cytometric analysis of the cells positively stained with Annexin V was performed using FACS Canto Flow Cytometer (BD Biosciences). The percentage of Annexin V-positive cells was analyzed with FlowJo Version 10 (TreeStar Inc., Ashland, OR, USA).

### Patients and tumor specimens

The children in this study were enrolled in Children's Oncology Group (COG) Neuroblastoma Biology Study 9047 through COG-affiliated locations, and were treated according to disease stage, age and tumor biology, as specified in institutional as well as different COG protocols [24]. The final cohort comprised 201 patients. The protocol was approved by individual institutional review boards, and informed consent was obtained for every patient registered in the study. TP53INP1 and N-Myc expression in the 201 human neuroblastoma samples was examined by real-time RT-PCR using TLDA microfluidic cards (Life Technologies, Grand Island, NY, USA).

TP53INP1 expression and N-Myc expression in neuroblastoma tissues were also analysed in 88 (Versteeg dataset) and 476 (Kocak dataset) human neuroblastoma samples in the publically available gene expression databases (http://r2.amc.nl). Clinical information for the 88 patients in the Versteeg dataset was directly down-loaded from http://r2.amc.nl, and clinical information for the 476 patients in the Kocak dataset was obtained from the authors' previous publications [25, 26].

### Statistical analysis

The mean ± standard error was calculated for each continuous variable of interest. Differences were tested for significance using ANOVA among groups or unpaired t-test for two groups. A probability value of 0.05 or less was considered significant. Pearson's correlation between TP53INP1 expression and N-Myc expression in the tumor tissues was calculated. The patient cohort was dichotomized into two groups (low versus high TP53INP1 expression) on the basis of the median value of TP53INP1 in the cohort, and repeated using the lower decile of TP53INP1 expression. Overall survival of patients was the time from diagnosis until death or until last contact if the patient did not die. Survival analyses were performed using GraphPad Prism 6.0 according to the method of Kaplan and Meier and comparisons of survival curves were made using two-sided log-rank tests [27].
